# Panaln: indexing pangenome for read alignment

**DOI:** 10.1093/bioinformatics/btaf476

**Published:** 2025-08-28

**Authors:** Lilu Guo, Zongtao He, Hongwei Huo

**Affiliations:** Department of Computer Science, Xidian University, Xi’an 710071, China; Department of Computer Science, Xidian University, Xi’an 710071, China; Department of Computer Science, Xidian University, Xi’an 710071, China

## Abstract

**Motivation:**

Pangenome indexing is a critical supporting technology in biological sequence analysis such as read alignment applications. The need to accurately identify billions of small sequencing fragments carrying sequencing errors and genomic variants drives the development of scalable and efficient pangenome indexing approach.

**Results:**

We propose a new wavelet tree-based approach, called Panaln, for indexing pangenome and introduce a batch computation approach for fast count query over Panaln. We present a simple and effective seeding strategy and develop a pangenome program that uses the seed-and-extend paradigm for read alignment. Experimental results on simulated and real data demonstrate that Panaln uses significantly less space for the compared pangenome methods with generally higher accuracy. We provide a scalable index construction by representing pangenome with a linear model. Additionally, Panaln brings enhanced accuracy compared to the popular single reference methods.

**Availability and implementation:**

Package: https://anaconda.org/bioconda/panaln and source code: https://github.com/Lilu-guo/Panaln.

## 1 Introduction

Ongoing advances in sequencing technology and bioinformatics algorithms are steering life sciences toward the ‘pangenome’ era. The term ‘pangenome’ denotes any collection of genomic sequences intended for joint analysis or use as the reference (Marschall *et al.* 2018). The use of pangenome can effectively address the incompleteness and bias limitations of the single reference genome methods, enhancing the accuracy of genome-wide association analysis (Valenzuela *et al.* 2018). Pangenome indexing is a core supporting tool in many sequence analysis tasks like read alignment (Eizenga *et al.* 2020, Liao *et al.* 2023). However, existing pangenome indexing methods require exponential construction space (Sirén *et al.* 2014, Kim *et al.* 2019) and the index itself occupies a huge space (Garrison *et al.* 2018, Rautiainen and Marschall 2020), which makes retrieval difficult. In recent years, aligning sequencing reads to the pangenome has gained wide attention, and developing a more efficient and scalable index for pangenome that enables fast retrieval in compact space is necessary and challenging (Sherman and Salzberg 2020, Baaijens *et al.* 2022).

To date, there are some impressive methods for pangenome indexing and read alignment that have been proposed. Here, we summarize them into three categories, described below.

1. Burrows-Wheeler (Burrows 1994) and FM-Index-based (Ferragina and Manzini 2000) graph indexing methods. They can support fast retrieval and encoding of single nucleotide variants (SNVs), insertion/deletion variants (INDELs), and structural variants (SVs). However, index construction of the Burrows-Wheeler-based graph is difficult because the number of potential paths increases exponentially with the number of variants (Huang *et al.* 2013). This results in suffix-based path sorting during index construction being highly demanding in both time and memory resources. Among them, GCSA (Sirén *et al.* 2014) generalizes the Burrows-Wheeler transform (BWT) and FM-Index to graphs, enabling the recognition of strings of any length corresponding to all possible paths through multiple alignments. GCSA2 (Sirén 2017) employs de Bruijn graphs as a *k*-mer index to alleviate the exponential space growth in GCSA by stopping index construction early. Consequently, the index accurately supports queries up to length *k*, while longer queries may yield false positives. VG (Garrison *et al.* 2018) uses GCSA2 to identify seeds and employs hash tables to index the variant graph structures, enabling versatile operations on graph with high memory usage. HISAT2 (Kim *et al.* 2019) uses a modified GCSA and hierarchical index, achieving fast query with small memory. Giraffe (Sirén *et al.* 2021) efficiently stores and queries thousands of haplotypes using the GBWT index (Sirén *et al.* 2020) and identifies seeds via a hash index. It is designed to map short reads to a pangenome reference.
2. *k*-mer-based graph methods. They store contiguous or overlapping genome fragments in each sequence node and link the nodes with edges. They can handle complex variants like duplications, inversions, and translocations. But they struggle with common small variants such as SNVs and INDELs due to the growth of nodes and edges. GraphAligner (Rautiainen and Marschall 2020) is a rapid hash-based long-reads aligner following the seed-and-extension paradigm. It employs a bit-parallel sequence-to-graph alignment algorithm (Rautiainen *et al.* 2019) to identify a path in the graph with minimum edit distance to the query sequence during the seed extension. Minigraph (Li *et al.* 2020) uses a minimizer-based (Roberts *et al.* 2004) hash index to construct a pangenome graph from dozens of human assemblies, compactly encoding SVs but not performing base alignment. Minichain (Chandra *et al.* 2024) is a novel haplotype-aware aligner that enhances sequence-to-graph chaining and alignment through recombination penalty for haplotype switches. It uses a hash index and solely supports SVs in the current proof-of-concept implementation, and its performance at the chromosome scale has not been explored.
3. Linear representation methods. They are easily scalable to the whole human genome and encode numerous SNVs and INDELs using a concise coordinate system. They can inherit the efficient search and alignment algorithms (Li and Durbin 2009, Marco-Sola *et al.* 2021) from classical methods, but are not suitable for representing complex SVs. BWBBLE (Huang *et al.* 2013) encodes reference genome and small variants by expanding nucleotide bases to include SNVs and appending padded gaps to linear representation sequence. vBWT (Iqbal *et al.* 2016) proposes positional markers to encode variants in a unified manner and uses a wavelet tree (Grossi *et al.* 2003) to cope with increased alphabet size. In addition, recent progress in compressed indexes (Huo *et al.* 2015, 2021, 2022, 2023, 2025a,b) provides new perspectives for pangenome indexing.

The wavelet tree (Grossi *et al.* 2003, 2011) is a powerful data structure. It can encode a text T of *n* symbols over an alphabet of size σ in $nH_{0}(T)+o(n\;\text{log}\;\sigma )$ bits of space while supporting efficient access in O(log σ) time, where $H_{0}(T)$ denotes the 0th-order empirical entropy of T (Manzini 2001). For a constant-sized alphabet, it supports constant-time access.

In this article, we introduce a wavelet tree-based method for efficient pangenome indexing using a linear representation model, propose an effective seeding strategy, and develop a pangenome aligner following the seed-and-extend paradigm.

## 2 Preliminaries

**Figure 1.**
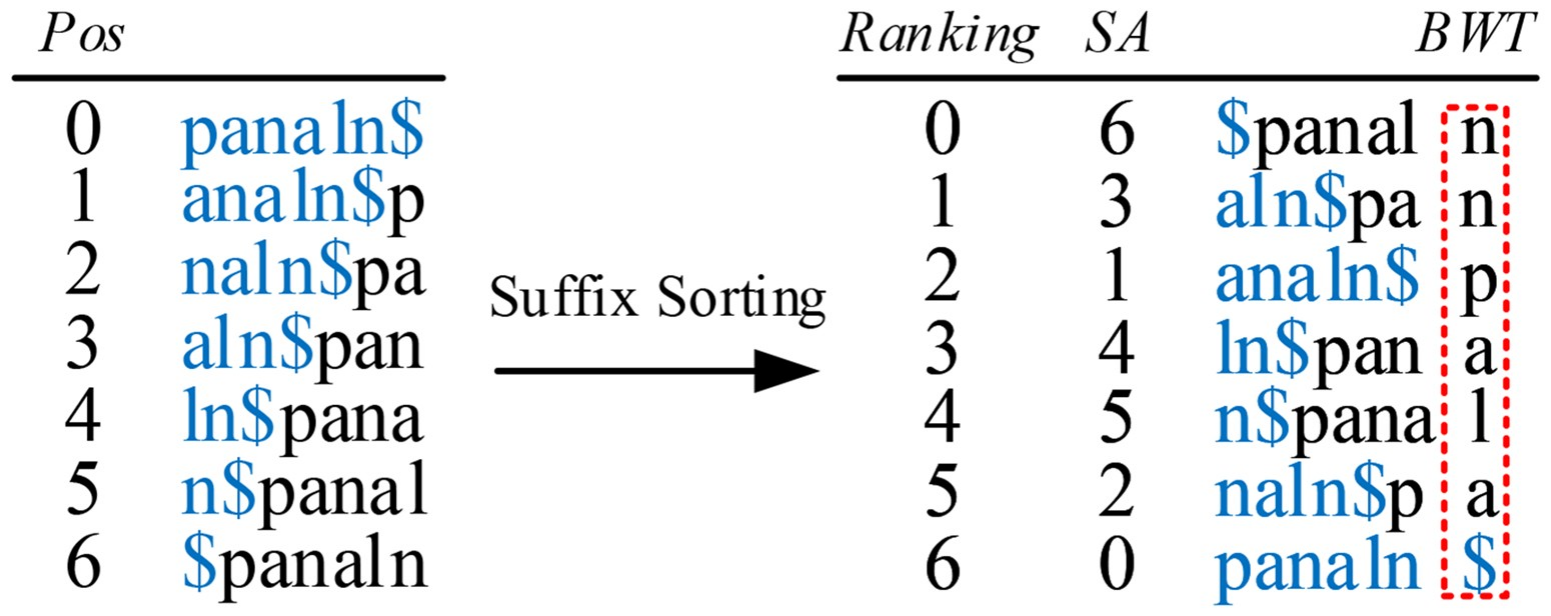
The Burrows-Wheeler transform for the text string T=panaln$.

FM-Index (Ferragina and Manzini 2000) is a high-order entropy-compressed text self-index based upon the BWT (Burrows 1994). For the text string T = T[0,n−1] over alphabet Σ, FM-Index can count the number of occurrences of a given pattern P in T in O(|P|) time. An example of the BWT is shown in Fig. 1 for T= panaln $, where the end-of-text symbol ‘$’ is lexicographically smaller than all other symbols in alphabet Σ. A suffix of T is a substring of the form T[j,n−1]. The suffix array *SA* (Manber and Myers 1993) of T gives the permutation of positions of all the suffixes in lexicographical order. The last column in Fig. 1 shows the *BWT* string of T.

Given a pattern P, all its occurrences in T form an interval [l(P),r(P)] in *SA*, where l(P) and r(P) are the indices in *SA* of the lexicographically smallest and largest suffixes prefixed with P. We can find the interval of P via *backward search* (Ferragina and Manzini 2000), which we show below.


(1) $$\{\begin{array}{c} l(cP^{\prime })=C[c]+Occ_{c}(\mathrm{BWT},l(P^{\prime }))\\ r(cP^{\prime })=C[c]+Occ_{c}(\mathrm{BWT},r(P^{\prime })) \end{array},$$

where C[c] stores the number of the symbols in T that are lexicographically smaller than *c*, and $Occ_{c}(\mathrm{BWT},i)$ answers the number of occurrences of *c* in *BWT[*0, *i]*. The mapping function *LF* is defined as $LF(c,i)=C[c]+Occ_{c}(\mathrm{BWT},i)$.

The wavelet tree is a binary tree where each node contains a bit string. Defined by recursively partitioning the alphabet into pairs of subsets, its leaves represent individual symbols of the alphabet, and each node’s bitvector stores whether a symbol of the string belongs to one subset or the other. It can efficiently answer $Occ_{c}(\mathrm{BWT},i)$ in entropy-compressed space. An example of wavelet tree is given in Fig. 1, available as supplementary data at *Bioinformatics* online.

Huo *et al.* (2015, 2025a) developed the practical FM-Index based upon BWT and wavelet trees, whose variant provides the foundation for indexing pangenomes.

The adoption of wavelet trees in FM-Index improves both space efficiency and query performance compared to data structures employed in other pangenome indexes. The fixed-length encoding is a common method for storing the *BWT* strings (Huang *et al.* 2013), and the space occupied by the sequence itself is n log σ bits. As $H_{0}(T)\leq \text{log}\;\sigma$, using the wavelet tree provides a more space-efficient alternative to the fixed-length encoding. In addition, the fixed-length encoding stores only a portion of the Occ(BWT,i) values, which necessitate the intra-block scanning. Due to the expansion of the pangenome alphabet, the intra-block scanning techniques (such as popCnt and Look-up table) become less efficient (Iqbal *et al.* 2016). While the wavelet tree can convert a query on strings to a set of constant-time rank queries on binary strings. Hash table is another commonly used data structure (Rautiainen and Marschall 2020, Sirén *et al.* 2021) that is accessed through a *k*-mer and stores the positions where the corresponding subsequence occurs in the genome or the read. This choice enables rapid *k*-mer/position correspondence at the cost of high memory usage. Another downside is that if a seed differs in a region between the reference and the query (e.g. due to an error or variant), there is no way to alternate the seeds in this region at mapping time (Sahlin *et al.* 2023). However, dynamic variable-length seeds are indexed in full-text data structures (e.g. suffix arrays or FM-Index), which can be computed on the fly at the mapping step and allow to find arbitrarily long queries in the genome.

## 3 Materials and methods

### 3.1 Indexing pangenome

#### 3.1.1 Pangenome representation

**Figure 2.**
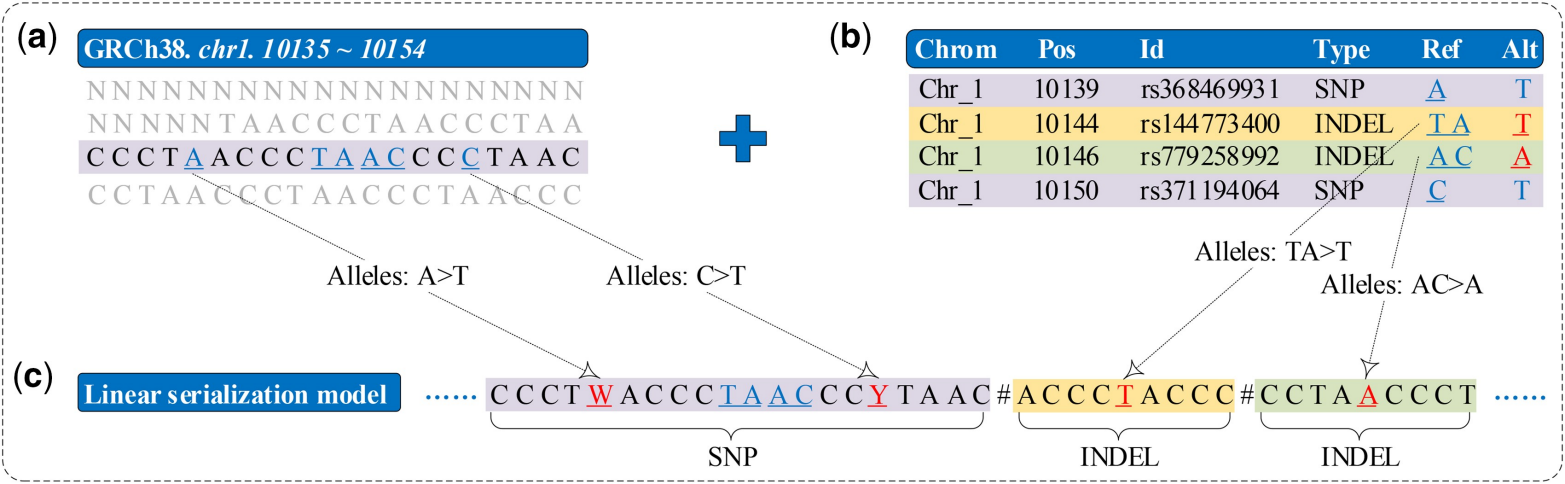
Linear representation sequence T = CCCTWACCCTAACCCYTAAC#ACCCTACCC#CCTAACCCT for the example pangenome shown in top of the graph, where (a) the reference genome, (b) the variation information, and (c) the pangenome representation.

We represent the pangenome using the method first developed by Huang *et al.* (2013), which combines a single reference genome as the backbone with a VCF (variant call format) file that provides observed population variants, such as the SNPs (single nucleotide polymorphisms) and INDELs (insertions and deletions). An example for pangenome representation is shown in Fig. 2, where the reference genome is illustrated in Fig. 2a and the SNPs and INDELs are depicted in Fig. 2b.

For SNPs, we extend the 4-symbol A/C/G/T nucleotide bases to 16-symbol IUPAC codes (Cornish-Bowden 1985) to accommodate allelic variants. This allows all observed SNPs from the VCF file to be encoded at specific positions on the backbone. For example, as shown in Fig. 2c, the underlined symbol ‘W’ at position 5 represents the allelic bases A/T, corresponding to the first item in the VCF file. The mapping relationship between IUPAC codes and the specific base combinations is defined in the first two rows of Table 1.

**Table 1.** Mapping of IUPAC to base combination and its complement.

IUPAC	T	K	G	S	B	Y	C	M	H	V	R	D	W	A
Base Comb.	T	GT	G	CG	CGT	CT	C	AC	ACT	ACG	AG	AGT	AT	A
Complement	A	M	C	S	V	R	G	K	D	B	Y	H	W	T

For INDELs, we concatenate each insertion and deletion variant from the VCF file with its flanking length-*k* context (a substring) in the genome, and then append them at the end of the backbone one by one, separated by symbol #. For example, the first INDEL fragment, as shown in Fig. 2c, corresponds to a sequence concatenated by Alt ‘T’ from the second item in the VCF file and length-4 context preceding and following it. The context size *k* is a tunable parameter preset to match the length of the sequencing reads.

We perform three additional processes. First, ambiguous bases ‘N’ in the reference genome are converted to bases A/C/G/T randomly as done in (Li and Durbin 2009). Second, we use the linear model (Huang *et al.* 2013) to represent the pangenome, and the result is denoted as T, as we show in Fig. 2. We assemble T and its reverse complement sequence together to form the final text. The last row of Table 1 shows the complement of IUPAC codes based on the combinations of nucleotide bases. Third, we construct an auxiliary structure *Ann* that records the start and end positions, which is used to convert the coordinates on padded INDELs from the pangenome representation sequence T to original chromosome sequence.

#### 3.1.2 Index construction

Let W be the *BWT* string of the pangenome representation sequence T over alphabet Σ, and let $\Sigma_{\text{uniq}}=\{A,C,G,T\}$ be the alphabet consisting of symbols that correspond to unique bases in W, and $\Sigma_{\text{poly}}=\{K,S,B,Y,M,H,V,R,D,W,\#,\$\}$ be the other alphabet consisting of symbols that map to polymorphic bases in W such that $\Sigma =\Sigma_{\text{uniq}}\cup \Sigma_{\text{poly}}$, where symbol ‘#’ is the separator and symbol ‘$’ is lexicographically smaller than all the other symbols in the alphabet Σ. Next, we will create a general wavelet tree over W in which the left subtree is a balanced wavelet tree (Foschini *et al.* 2004, Grossi *et al.* 2011) with the symbols from $\Sigma_{\text{uniq}}$ in W and the right subtree is a Huffman-shaped wavelet tree (Foschini *et al.* 2004, Grossi *et al.* 2011) with the remaining symbols from $\Sigma_{\text{poly}}$ in W.

First of all, we create a bit vector U with the length of |W| such that U[i]=1 if $W[i]\in \Sigma_{\mathrm{poly}}$ and U[i]=0 if $W[i]\in \Sigma_{\text{uniq}}$. Correspondingly, we concatenate the symbols in W in the same order such that $W[i]\in \Sigma_{\text{uniq}}$ to form a sequence $W_{l}$, represented as the balanced wavelet tree, and the remaining symbols in W in the same order to form a sequence $W_{r}$, represented as the Huffman-shaped wavelet tree. The *BWT* string W corresponding to the linear representation sequence T for the example pangenome shown in Fig. 2c is TCCTTTATAW#AACCAAAA$AC#CCCCCCCCCCCYCCCCTC. The bit vector U is 00000000011000000001001000000000001000000, $W_{l}$ = TCCTTTATAAACCAAAAACCCCCCCCCCCCCCCCTC, and $W_{r}$ = W#$#Y, respectively.

**Figure 3.**
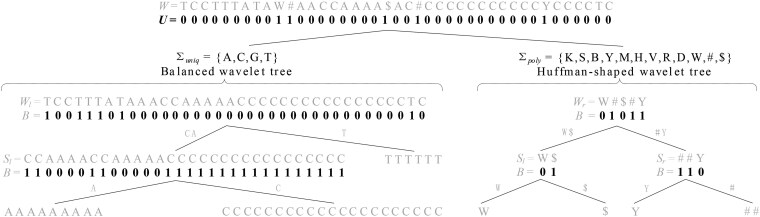
Wavelet tree-based structure for pangenome index.

As shown in Fig. 3, we construct a balanced wavelet tree for the sequence $W_{l}$ and a Huffman-shaped wavelet tree for the sequence $W_{r}$. This is motivated by the distribution of symbols in the pangenome, i.e. $W_{l}$ has a small alphabet $\Sigma_{\text{uniq}}$ with uniform distribution, while the more symbols in $W_{r}$ are non-uniformly distributed. As $|W_{l}|:|W_{r}|\approx 210:1$, $W_{r}$ is much shorter than $W_{l}$. For the human pangenome, the Huffman-shaped wavelet tree for $W_{r}$ is small in size, a few dozen megabytes, allowing it to be loaded into the cache memory for fast access.

This design avoids the inefficiency of $Occ_{c}(S,i)$ query for the symbol set $\Sigma_{\text{poly}}$, which would otherwise require random access with low cache hits on a wavelet tree of nearly two gigabytes in size, where all symbols are mixed. For the $\Sigma_{\text{uniq}}$, this way also reduces the tree height and speeds up the query. In addition, we construct a compact structure for bit vector U while supporting efficient ${\text{rank}}_{1}(U,i)$. The details of compact structure and access are given in Table 1, available as supplementary data at *Bioinformatics* online.

To answer the $Occ_{c}(W,i)$ query on our wavelet tree-based structure, we perform the following steps: if U[i]=0, we set $i\leftarrow i-{\text{rank}}_{1}(U,i)$ and access the balanced wavelet tree; otherwise, we set $i\leftarrow {\text{rank}}_{1}(U,i)$ and access the Huffman-shaped wavelet tree. After this, we perform the $Occ_{c}(S,i)$ operation on either the balanced wavelet tree with $S=W_{l}$ or the Huffman-shaped wavelet tree with $S=W_{r}$. Algorithm 1, available as supplementary data at *Bioinformatics* online provides the computation for the $Occ_{c}(S,i)$ operation.

#### 3.1.3 Fundamental queries

We introduce three fundamental queries with the pangenome index: count, locate, and extract. The count query reports the number of occurrences in the text T of pattern P, the locate query locates the starting positions in text T where pattern P occurs, and the extract query retrieves the length-ℓ substring T[pos,pos+ℓ−1] starting from a given position *pos*.

Let $\Gamma_{\gamma }$ denotes the subset of the IUPAC code listed in Table 1 that contains a specific base γ∈{A,C,G,T}, as done in Huang *et al.* (2013). Specifically, $\Gamma_{A}=\{M,H,V,R,D,W,A\}$, $\Gamma_{C}=\{S,B,Y,C,M,H,V\}$, $\Gamma_{G}=\{K,G,S,B,V,R,D\}$, and $\Gamma_{T}=\{T,K,B,Y,H,D,W\}$. For a DNA query pattern P, it is likely to have different match substrings in the pangenome (for instance, P = *TAA* is compatible with strings like *TAA*, *TWA*, etc.), meaning it may align to multiple intervals in *SA*. So we use S to denote the set of these intervals. Algorithm 2, available as supplementary data at *Bioinformatics* online provides the pseudocode for the count query on the pangenome, in which the **for** loop in lines 2–10 is a variation of the *backward search*, which considers the subset $\Gamma_{\gamma }$ of the IUPAC code, and keeps track of the multiple SA intervals in S.

As shown in the count pseudocode of line 5 in Algorithm 2, available as supplementary data at *Bioinformatics* online, $Occ_{\phi }(W,i)$ is a critical low-level operator used to support the calculation of the *LF* mapping function within our pangenome indexing. Formally, let W be the *BWT* string over the alphabet Σ, given query position i≤|W| and symbol set ϕ⊆Σ, the $Occ_{\phi }(W,i)$ is to calculate the number of occurrences of each c∈ϕ in *BWT[*0, *i]*, where ϕ is dynamically assigned depending on the specific query base in the read sequence.

An efficient batching method is proposed to calculate the $Occ_{\phi }(W,i)$ operator for assigned symbol set ϕ in a one-pass way. However, the naive method of calling $Occ_{c}(S,i)$ operation requires multiple queries to get the value of each symbol c∈ϕ. Essentially, the calculation of $Occ_{c}(S,i)$ by using the wavelet tree is implemented through a series of ${\text{rank}}_{b}(B,i)$ operations from the root node to leaf nodes. Therefore, there are typically some common ancestor nodes starting at the root node among different symbols at the leaf nodes. By sharing the ${\text{rank}}_{b}(B,i)$ operations on these common ancestor nodes through a batch method, the efficiency can be improved. The pseudocode for the batching query $Occ_{\phi }(W,i)$ is provided in Algorithm 3, available as supplementary data at *Bioinformatics* online. Here, ϕ represents the assigned symbol set to be queried, A and $A^{\prime }$ store the *Occ* values, $\Sigma_{l}$ denotes the first part of the alphabet symbols at the current node, and $\Sigma_{r}$ denotes the remaining alphabet symbols. A further illustration of the operator $Occ_{\phi }(W,i)$ is shown in Fig. 2, available as supplementary data at *Bioinformatics* online.

We describe the locate query with the pangenome index in Algorithm 4, available as supplementary data at *Bioinformatics* online, which returns the chromosome ID and the position where the pattern occurs. The pseudocode for the extract query is described in Algorithm 5, available as supplementary data at *Bioinformatics* online. Given the starting position *pos* and query length ℓ, it extracts the corresponding substring from the pangenome index.

### 3.2 Pangenome read alignment

In this section, we first introduce the concept of longest equal overlapping fragment (LEOF) and propose an effective seeding strategy based on this concept in Section 3.2.1. Then we define seed as the fragment in a read that corresponds to LEOF and propose a pangenome tool for read alignment in Section 3.2.2.

#### 3.2.1 Finding the LEOF

**Figure 4.**
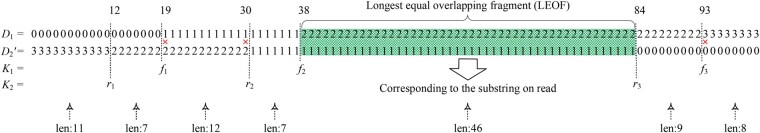
Finding the LEOF using D arrays.

Before we introduce the LEOF, we first give the description of D array (Li and Durbin 2009). The D array is a continuously increasing integer array (see Fig. 4) of read R, and its length is equal to |R|. The value of D[i] indicates the minimum number of fragments that the substring R[0,i] should be partitioned into, to ensure that each fragment can occur at least once in the reference genome. That is, this value serves as a lower bound for the number of differences within the substring R[0,i]. The D array was previously used for space pruning in backtracking search (Canzar and Salzberg, 2017), and here we generalize it to the pangenome and use it to find variable-length seed on the read sequence. Specifically, our focus is on the location of discontinuity points in the D array. These points help estimate the possible locations of differences (i.e. sequencing errors or unknown variants) between the read and its source fragment.

Definition 1 (Equal Overlapping Fragment).

*Given a read* R  *and its reverse complement sequence* $\bar{R^{{}^{\prime }}}$*, let* $D_{1}$  *and* $D_{2}$  *denote the* D  *arrays of* R  *and* $\bar{R^{{}^{\prime }}}$*, and let* $D_{2}^{{}^{\prime }}$  *be the reversed array of* $D_{2}$*. Let* $K_{1}$  *be the position set of discontinuous points in* $D_{1}$  *such that* $D_{1}[i]\neq D_{1}[i+1]$  *and let* $K_{2}$  *be the position set of discontinuous points in* $D_{2}^{{}^{\prime }}$  *such that* $D_{2}^{{}^{\prime }}[i]\neq D_{2}^{{}^{\prime }}[i+1]$*. We define the Equal Overlapping Fragments (EOF) as fragments on* R  *separated by the merge-sorted positions of* $K_{1}$  *and* $K_{2}$*. LEOF is the longest fragment among all the EOFs in* R.

An example of the D arrays and EOFs is shown in Fig. 4. The given read contains three sequencing errors at locus 19, 30, and 93, marked with a red cross. The positions sets $K_{1}=\{f_{1},f_{2},f_{3}\}$ and $K_{2}=\{r_{1},r_{2},r_{3}\}$. According to Definition 1, we can get seven EOFs with different lengths (i.e. fragments separated by the dashed lines); the length of LEOF is 46.

The idea of EOF is to identify the positions of differences by leveraging the intrinsic information of the paired D arrays. As estimated identical fragments between the read and its source fragment on the genome, EOFs are considered to be located in the region between differences on the read. Algorithm 6, available as supplementary data at *Bioinformatics* online describes the pseudocode of finding LEOF.

Essentially, our strategy involves dividing the read sequence into a set of fragments with no differences using the paired D arrays. To achieve this goal, there are two problems that need to be addressed. Firstly, the positions of the discontinuity points in D arrays are often behind the true position where the difference occurs, which is the ‘delay phenomenon’. For example, the difference at locus 30 in Fig. 4 is delayed by eight bases before being reflected on the discontinuity point at locus 38. Therefore, we avoid this by combining $D_{1}$ and $D_{2}$ arrays. Secondly, some short EOFs with differences may happen to match to other places in the genome that are not their true source, which is the ‘false positive phenomenon’. Therefore, we use the longest EOF to improve uniqueness and sensitivity. As an estimated seed, LEOF is designed to find a few valid candidate hits in the genome where the read is likely to align.

#### 3.2.2 LEOF-based search

**Figure 5.**
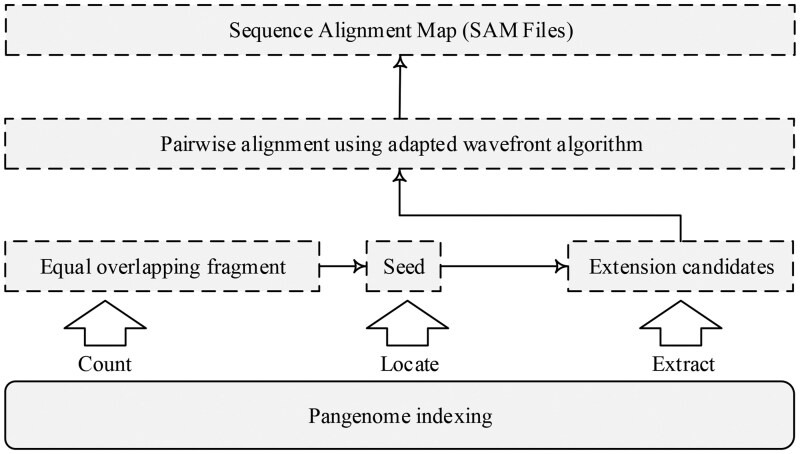
The framework of pangenome read alignment.

We designed an LEOF-based pangenome program for read alignment applications under the seed-and-extend paradigm. This program utilizes the three fundamental pattern queries: count, locate, and extract, provided by the pangenome index, and integrates the adapted wavefront algorithm (WFA) for pairwise alignment. Its framework is shown in Fig. 5. Pangenome indexing, the core engine of this system, provides a compact index structure and efficient data retrieval. Firstly, we use the count query provided by the pangenome index (see Algorithm 2, available as supplementary data at *Bioinformatics* online) to calculate D arrays and then derive LEOF. Next, LEOF is used as a seed and located using the locate query to determine its position on the pangenome. Finally, we use the extract query to retrieve the flanking sequence around the seed position from the pangenome index as extension candidates (see Guo and Huo 2024 for details).

Next, we perform pairwise alignment between extension candidates and the read sequence to identify the best candidate and base-level alignment. To the best of our knowledge, WFA is one of the fastest gap-affine pairwise alignment algorithms utilizing wavefront alignment in most scenarios. Different from the traditional dynamic programming algorithms that require quadratic time on the length of the sequences, the complexity of the wavefront algorithm is O(ns), where *n* is the sequence’s length and *s* is the alignment score (Marco-Sola *et al.* 2021, 2023). Note that WFA finds the minimum-cost alignment by using a positive penalty, working as a minimization problem. In our pangenome, the observed multiple alleles are coded as IUPAC, e.g., ‘W’ means ‘A’ or ‘T’. Therefore, we further modified the wavefront extend function of WF_EXTEND in WFA (see Algorithm 2 of the work (Marco-Sola *et al.* 2021) for details) to replace the explicit character comparison with this inclusive relationship mapping. This redesign enables the adapted WFA to be used for pangenome alignment.

It brings the following benefits: (1) faster speed compared to the backtracking search of extending alignments one by one in BWBBLE; (2) less memory usage compared to the explicit storage of genome in a 2-bits-per-base encoding in HISAT2, as extracting extension candidates from the index structure can significantly save memory; and (3) competitive accuracy, because variants are fully retained during both the seed and extension, ensuring the completeness and unbiasedness of the reference.

## 4 Results

In this section, we present an evaluation of Panaln using both simulated and real data to compare its accuracy, memory usage, and speed with respect to the popular read alignment approaches (Alser *et al.* 2021), namely, Bowtie2 (v2.4.5) (Langmead and Salzberg 2012), BWA-MEM (v0.7.17) (Li 2013), BWA-MEM2 (v2.2.1) (Vasimuddin *et al.* 2019), BWBBLE (Huang *et al.* 2013), and HISAT2 (v2.2.1) (Kim *et al.* 2019). Among them, the first three methods are for the single reference genome, and the latter two are for pangenomes. In addition, we give the detailed commands of each comparison method used in the experiment in Table 3, available as supplementary data at *Bioinformatics* online.

**Table 3.** Performance of accuracy and speed on simulated reads.

# sim_MS	Cor. mapped	Mapped	Run. time
Panaln	95.6481%	100.0%	5971 s
BWBBLE	95.7087% (0.0606%) ↑	99.98%	28 511 s
HISAT2	95.5158% ↓ (0.1323%)	99.72%	180 s
Bowtie2	94.7760% ↓ (0.8721%)	99.97%	449 s
BWA-MEM	95.0928% ↓ (0.5553%)	100.0%	377 s
BWA-MEM2	95.0928% ↓ (0.5553%)	100.0%	164 s

# sim_S	Cor. mapped	Mapped	Run. time
Panaln	95.7858	100.0%	3413 s
BWBBLE	95.7844% ↓ (0.0014%)	99.98%	23 468 s
HISAT2	95.6660% ↓ (0.1198%)	99.76%	119 s
Bowtie2	94.9389% ↓ (0.8469%)	99.99%	425 s
BWA-MEM	95.1401% ↓ (0.6457%)	100.0%	315 s
BWA-MEM2	95.1401% ↓ (0.6457%)	100.0%	130 s

# sim_M	Cor. mapped	Mapped	Run. time
Panaln	95.4676	100.0%	5914 s
BWBBLE	95.5358% (0.0682%) ↑	100.0%	26 838 s
HISAT2	95.4802% (0.0126%) ↑	99.78%	158 s
Bowtie2	95.7091% (0.2415%) ↑	100.0%	395 s
BWA-MEM	95.7268% (0.2592%) ↑	100.0%	262 s
BWA-MEM2	95.7268% (0.2592%) ↑	100.0%	123 s

### 4.1 Experimental setting and data

All experiments were run on a Dell T7910 workstation with a Dual Intel Xeon E5-2650 CPU at 2.20 GHz, 30 MB Caches, 198 GB DDR4 RAM, and 4 TB SATA HDD. The operating system is Ubuntu 20.04.6 LTS 64-bit; our program was implemented by C mixed with C++ and compiled using GCC v9.4.0 with the -O3 compilation optimization option.

The human GRCh38 is used as the reference genome. For the simulated reads, we use the built-in simulator (https://github.com/DaehwanKimLab/HISAT2/blob/master/) of HISAT2 to generate three simulated datasets from the reference genome plus the common small variants (http://hgdownload.soe.ucsc.edu/goldenPath/hg38/database/) (including SNP, insertion, and deletion) in dbSNP (v144) database. Each of the simulated datasets contains one million 101-bp Illumina-like paired-end reads with different parameter settings: (i) sim_MS, reads including known variants with 0.2% per-base sequencing errors; (ii) sim_S, reads including known variants with no sequencing errors; and (iii) sim_M, reads with 0.2% per-base sequencing errors and no known variants. Meanwhile, the simulator outputs the corresponding sam file as ground truth to check the correctness. For the real reads, we first download the 250-bp Illumina reads dataset of the public sample HG004 with whole-genome sequencing, as well as the gold standard SNVs and INDELs, which are provided by the Genome-IN-A-Bottle (GiaB) consortium. Considering the execution time of subsequent variant calling, we use the first one million subsets of the full dataset with 17× coverage in the experiment. Then, we download the PacBio-CCS reads dataset of the sample NA24385 from NCBI. They are variable-length sequences with an average length of about 10k bp. In the experiment, we fetch the first 10 000 reads for benchmarking.

### 4.2 Performance evaluation

For each method, the index has been constructed in advance, and the same set of common small variants in the dbSNP (v144) database is used to construct the pangenome index. In the experiments, we ran each program ten times and took the average running time, which refers to wall clock time. Before each run, the cache data in memory is cleaned to avoid the impact of index loading. Memory usage refers to the maximum amount of resident memory used during program running. In addition, we evaluated the accuracy of each method concerning two aspects. The first is the proportion of overall mapped reads in the input reads. The second is the proportion of correctly mapped reads in the input reads; we consider a read to be correctly mapped according to the alignment file generated by the simulator if the reported alignment overlaps with the simulator-provided region by at least 90%. This accuracy measure was also used by HISAT2.

#### 4.2.1 Performance of scalability on index construction

HISAT2 is an ultra-fast and highly optimized pangenome approach that relies on a prefix-sorted automaton, drawing some inspiration from GCSA (Sirén *et al.* 2014). However, its index construction algorithm has exponential complexity, resulting in significant memory usage and running times to construct the index. For comparison with HISAT2, we conduct experiments using the same pruned variants from dbSNP that have been preprocessed by HISAT2’s script. Additionally, we test the full variants to evaluate the scalability of each pangenome method in terms of time and memory usage.

Table 2 shows the performance of scalability over index construction, where $n_{1}$ and $n_{2}$ represents the number of SNPs and INDELs, and the notation ‘-’ denotes index construction or program failure (same as below). We observed that HISAT2 fails to construct the pangenome index, even if we use another server with 1 TB RAM. Using haplotype alleviates the memory requirement, but it still consumes considerable space and time.

**Table 2.** Performance of scalability over pangenome index construction.^a^

Variant set	Method	Mem. usage	Con. time
dbSNP-Pruned	Panaln	63 GB	48 min
$n_{1}$ = 12 921 989	BWBBLE	59 GB	86 min
$n_{2}$ = 1 538 418	HISAT2	–	–
	HISAT2 + $\mathrm{haplotyp}e^{*}$	171 GB	525 min
dbSNP	Panaln	114 GB	94 min
$n_{1}$ = 133 818 343	BWBBLE	105 GB	136 min
$n_{2}$ = 11 989 100	HISAT2	–	–
	HISAT2 + $\mathrm{haplotyp}e^{*}$	–	–

a $\mathrm{haplotyp}e^{*}$ : This extra file is not necessary for HISAT2, but haplotype information can keep the index construction from exploding.

#### 4.2.2 Performance on simulated reads

The source location and true alignment of each read in the simulated dataset are known. This enables us to gain a deeper understanding of the correctly mapped ratio and alignment of each method on simulated reads under different settings.

For the sim_MS dataset, it can be concluded from Table 3 that BWBBLE and Panaln have competitive and relatively close accuracy. However, in terms of speed, Panaln is 4.8× times faster than BWBBLE. We also observed that although Bowtie2 and BWA-MEM have competitive overall mapped ratios, their correctly mapped ratios are relatively lower, indicating a higher incidence of false positive errors. At the same time, the accuracy of pangenome methods (including Panaln, BWBBLE, and HISAT2) is generally higher than that of single reference genome methods (including Bowtie2, BWA-MEM, and BWA-MEM2), while the speed tends to be slower due to the expansion of the reference space. HISAT2, as a special case, provides ultra-fast speed, even outperforming those approaches designed for single reference genomes. This exceptional performance in speed is largely attributed to its BWT-based graph index, at the expense of throwing the heavy computation to the index construction.

For the sim_S dataset, we can observe that Panaln has achieved higher accuracy and speed here. This is because the reference biases caused by individual genetic variants can be effectively reduced using the pangenome. In addition, since known genetic variants have been included in the pangenome index, the LEOF-based search here is actually simplified to an exact match, so the running speed is faster than that on the sim_MS dataset. Furthermore, we can see that Bowtie2 and BWA-MEM have a higher correctly mapped ratio than pangenome methods from the sim_M dataset. One reason is that there are just sequencing errors without any reference bias caused by genetic variants, so it cannot take advantage of the efficacy of the pangenome. Another reason is that the additional variant information incorporated in the pangenome expands the reference space and increases the possibility of false positives by introducing more fake candidate alignments. However, both the sim_S and sim_M datasets are the special comparison cases, which are used to investigate and analyse the characteristics of the pangenome. Relatively speaking, the sim_MS dataset is closer to the characteristics of the real-world reads and is thus more valuable for performance evaluation.

#### 4.2.3 Performance on real reads

The source location and alignment of biological reads output by the sequencer are typically unknown. Fortunately, the GiaB consortium provides a gold standard of the individual genetic variants in a few samples for the research community. This allows us to quantify the performance of each method on real reads and assess their accuracy in downstream variant calling.

Table 4 shows the performance of five comparison methods and Panaln on real reads. Both Bowtie2 and Panaln have small space usage, with index sizes less than 4 gigabytes. Among the pangenome methods, the index space usage of Panaln is 31% of BWBBLE and 54% of HISAT2 (see Table 4, available as supplementary data at *Bioinformatics* online for detailed index space usage of Panaln). For Illumina reads, Panaln achieved the highest mapped ratio, while HISAT2 had the fastest running speed. Notably, to achieve a comparable mapped ratio, we adjusted BWBBLE’s *k*-difference option to 7 to accommodate longer length of the reads provided by the GiaB consortium. For PacBio-CCS reads, BWA-MEM’s chain-based search method performed exceptionally well due to its suitability for long reads. BWBBLE was unable to process long reads due to a segmentation fault. HISAT2’s mapped ratio was less competitive, and Panaln’s search strategy requires refinement to better adapt to the features of long reads.

**Table 4.** Performance of space usage and speed on real reads.^a^

Method	Index size	Illumina		PacBio-CCS	
		Mapped	Time	Mapped	Time
Panaln	3.7 GB	99.95%	18315 s	98.94%	1993 s
BWBBLE	12.0 GB	90.16%	78802 s	–	–
HISAT2	6.8 GB	92.72%	263 s	89.77%	300 s
Bowtie2	2.9 GB	96.76%	1148 s	94.61%	86 592 s
BWA-MEM	5.2 GB	99.51%	899 s	99.91%	1185 s
BWA-MEM2	16.2 GB	99.51%	631 s	99.91%	1622 s

a The default settings for the source code of BWA-MEM2: SA sampling rate is 8, and compression factor η is 64.

To evaluate the confidence of Panaln in downstream tasks, we built a complete variant calling pipeline, presented the SNV and INDEL variants called by Panaln, and compared them to other aligners. Following the pipeline described in previous work (Sahlin 2022), we used bcftools to benchmark the *F*-score, precision, and recall of SNV and INDEL calling over each aligner’s output on Illumina real reads provided by the GiaB consortium. The GitHub repository for GiaB offers a public truth set that is continuously updated using various sequencing platforms. The gold standard truth set for validation includes 3,453,580 SNVs and 577,766 INDELs.

The results of SNV and INDEL calling with each method on the GiaB dataset can be seen in Table 5, available as supplementary data at *Bioinformatics* online. We denote the number of variants detected by the aligner as P, the number of true variants provided by the GiaB gold standard as T, and the number of variants obtained by taking the intersection of the variants detected by the aligner and the true variants provided by the GiaB gold standard as TP. As stated in the literature (Yan *et al.* 2021, 2022), the precision is calculated as TP/P, the recall is calculated as TP/T, and the *F*-score is the harmonic mean of precision and recall. Note that the poor results of all tools are due to the low coverage of the reads dataset. As mentioned in the literature (Sirén *et al.* 2021), higher coverage can produce better variant calls; however, the read coverage in this experiment is fair and unbiased for all compared methods. For SNV calling, HISAT2 has the highest recall, i.e. it finds the most true positive variants, followed by Panaln. However, the precision of HISAT2 is much lower than that of Panaln, which makes Panaln achieve the highest *F*-score. For INDEL calling, Panaln has the highest *F*-score compared to other methods. BWA-MEM, as the tried-and-tested single reference genome method, has competitive performance in variant calling.

**Table 5.** Comparison with pangenome aligners on human chromosome 1.

Method	Index construct		Run.m	L101_E0.2		L250_E0.2		L101_E0.1		L250_E0.1		CCS_10k_Q20		CCS_10k_Q30	
	Cons.t	Cons.m		Cor.m	Time	Cor.m	Time	Cor.m	Time	Cor.m	Time	Cor.m	Time	Cor.m	Time
Panaln	4 m	5.3 GB	0.29 GB	96.96	2870	98.47	5273	97.00	2542	98.57	4287	99.43	3041	99.47	1425
BWBBLE	6 m	4.9 GB	0.95 GB	96.97	13318	98.49	25521	97.00	13080	98.50	22938	–	–	–	–
VG	1 h:13 m	27.4 GB	2.10 GB	90.92	2358	95.54	3389	90.94	2315	95.85	3356	99.30	10912	99.36	5770
HISAT2	1 h:50 m	67.3 GB	0.90 GB	96.87	140	98.54	187	96.93	134	98.57	172	97.24	650	99.57	50
GraphAligner	4 m	9.6 GB	5.00 GB	82.48	2692	97.95	6709	58.65	2504	97.96	6782	99.93	3043	99.97	2824
Giraffe	12 m	9.2 GB	3.30 GB	97.00	625	98.74	1119	97.03	616	98.75	1104	16.08	301	38.50	776

The underlined value indicates the best result among all methods for that metric column.

#### 4.2.4 Extension comparison with pangenome aligners

We compared our approach with more existing pangenome aligners, including VG (v1.63.1), GraphAligner (v1.0.20), Giraffe (v1.63.1), and Minichain (v1.3), etc. Note that Giraffe has been integrated into the VG toolkit as a module, so here they share the same version number. In our experiments, VG refers to the VG-MAP module by default unless otherwise specified. Minichain’s current proof-of-concept solution failed to perform the chromosome-scale pangenome alignment. We also investigated MiniGraph (Li *et al.* 2020) and tried to compare it with us, but its input depends on a series of human assemblies; we cannot generate its pangenome using the given reference sequence and variants in the dbSNP database.

Burrows-Wheeler graph-based methods HISAT2 and VG have considerable memory and disk space requirements for computing devices when constructing whole-genome indexes. To this end, we follow the experimental design in the literature (Rautiainen and Marschall, 2020) of GraphAligner and use the chromosome 1 reference (GRCh38) and the corresponding variants in the dbSNP database to generate pangenome, where the length of chromosome 1 is 248,956,422, the number of SNVs is 971,371, and the number of INDELs is 116,943. It should be noted that the FASTA file size for chromosome 1 is 242MB, the largest of all human chromosomes. In the experiment, we generated six simulated datasets from the human chromosome 1 reference, incorporating known variants from the dbSNP database. These datasets, varying in read lengths and error rates, are named L101_E0.2, L250_E0.2, L101_E0.1, L250_E0.1, CCS_10k_Q20, and CCS_10k_Q30. Here, ‘L’ denotes the read length and is set to 101-bp or 250-bp, and ‘E’ denotes the base error rate and is set to 0.2% or 0.1%. The first four datasets each contain 2 million Illumina-like reads, and the latter two datasets each contain 20 000PacBio-CCS-like reads of 10k-bp in length, with the error rate of 1% (i.e., Q20) and 0.1% (i.e. Q30), respectively.

Table 5 shows the comprehensive results of the comparison to state-of-the-art pangenome aligners, including construction time (abbreviated Cons.t) and the peak construction memory (abbreviated Cons.m) for the index construction, as well as the running time for read alignment, the correctly mapped ratio (abbreviated Cor.m) and the peak running memory (abbreviated Run.m) during alignment. In terms of the index construction, VG and HISAT2, as the Burrows-Wheeler-based graph methods, require significantly more construction time and memory, usually several to dozens of times that of other pangenome methods. This is because their suffix-based path sorting algorithm consumes lots of space and time, especially when the number of paths on the graph grows exponentially with the encoded variants. In addition, because GraphAligner, Giraffe, and VG use hash tables to index the graph structures, they require significantly more memory in read alignment than other compressed index-based pangenome methods such as Panaln, BWBBLE, and HISAT2. In terms of the correctly mapped ratio, Giraffe performs best with Illumina short reads, GraphAligner excels with PacBio-CCS long reads, and Panaln demonstrates highly competitive accuracy, nearly matching the top method, across both Illumina and PacBio-CCS reads.

## 5 Discussion and limitation

In this study, we propose an alternative solution that enhances retrieval efficiency and construction scalability in pangenome indexing. Our approach Panaln introduces a new wavelet tree-based method for indexing pangenome, and proposes a batch computation approach for fast count query on pangenome. Furthermore, Panaln uses an estimated variable-length seed of LEOF and an adapted wavefront algorithm for read alignment tasks. Extensive experiments confirm the competitiveness of Panaln in space usage and accuracy. The linear representation adopted in Panaln provides lightweight and scalable index construction; however, it makes Panaln less competitive with the Burrows-Wheeler-based graph indexing methods in speed.

## Supplementary Material

btaf476_Supplementary_Data

## Data Availability

The real data used in this article are available through the Genome-IN-A-Bottle (GiaB) consortium at https://ftp-trace.ncbi.nlm.nih.gov/ReferenceSamples/giab and can be accessed using the download link provided in the Supplementary Material at *Bioinformatics* online.
