# Supplementary material for: Panaln: indexing pangenome for read alignment
Source: Bioinformatics. 2025 Aug 28;41(9):btaf476. doi: 10.1093/bioinformatics/btaf476 (PMC12448906; doi:10.1093/bioinformatics/btaf476)
Supplement: btaf476_Supplementary_Data [file btaf476_supplementary_data.pdf]

SUPPLEMENTARY INFORMATION

# PanaIn: Indexing pangenome for read alignment

**Lilu Guo, Zongtao He, and Hongwei Huo***

Department of Computer Science, Xidian University, Xi'an, Shaanxi, 710071, China

*To whom correspondence should be addressed.
Email: hwhuo@mail.xidian.edu.cn

We start to show the benefits of wavelet tree over other solutions. The wavelet tree [3, 4] is a versatile and powerful data structure. It can encode a text $\mathcal{T}$ of $n$ symbols over alphabet $\Sigma$ of size $\sigma$ in entropy-compressed space of $n\mathcal{H}_0(\mathcal{T}) + o(n \log \sigma)$ bits while supporting efficient access in $\mathcal{O}(\log \sigma)$ time, where $\mathcal{H}_0(\mathcal{T})$ denotes the 0th-order empirical entropy of $\mathcal{T}$ [15]. For constant-sized alphabets, it supports constant-time access.

Fixed-length encoding is a widely used method for storing $BWT$ strings [5, 13, 12, 11], and the space occupied by the sequence itself is $n \log \sigma$ bits. Because theoretically $\mathcal{H}_0(\mathcal{T}) \leq \log \sigma$, using wavelet tree brings a space benefit when compared to fixed-length encoding. In addition, only a portion of $Occ(BWT, i)$ are stored in this method, the intra-block scanning is required. However, as the alphabet in pangenome increases from 4-symbol A/C/G/T in the single reference genome to 16-symbol IUPAC code, intra-block scanning techniques (such as popCnt and Look-up table) become inefficient [10]. The wavelet tree, a binary tree where each node contains a bit string, transforms a query on string into a set of constant-time rank queries on bit strings, offering an efficient solution for pangenome indexing.

Hash table is another commonly used data structure [16, 2, 19, 14] that is accessed through a $k$-mer and stores the positions where the corresponding subsequence occurs in the genome or the read. This choice allows a very fast $k$-mer/position correspondence, but is memory-wise costly as it implies that the position value of the hash table are not compressed [1]. Another downside is that if a seed differs in a region between the reference and the query (e.g., due to an error or variant), there is no way to alternate the seeds in this region at mapping time [18]. However, dynamic variable-length seeds are indexed in full-text data structures (e.g., suffix arrays or FM-Index), which can be computed on the fly at the mapping step and allow to find arbitrarily long queries in reference genome.

**Algorithm 1:** $Occ_c(S, i)$
$root$ is the root node of the wavelet tree for text string $S$, $\Sigma_l$ is the alphabet of left subtree, $\mathcal{B}$ is the bit string at a node.

```
node ← root
while ! node.isLeaf do
    if c ∈ node.Σ_l then
        i ← i − rank_1(node.B, i)
        node ← node.lChild
    else
        i ← rank_1(node.B, i)
        node ← node.rChild
return i
```

**Supplementary Algorithm 1**

To answer the $Occ_c(S, i)$ operator, we start at the root node and perform the following steps. If $c \in \Sigma_l$, we set $i \leftarrow \text{rank}_0(\mathcal{B}_{root}, i)$ and move to the left child; otherwise, we set $i \leftarrow \text{rank}_1(\mathcal{B}_{root}, i)$ and move to the right child. Here, $\text{rank}_b(\mathcal{B}, i)$ returns the number of occurrences of $b$ in $\mathcal{B}[0, i]$ for $b \in \{0, 1\}$. We iterate these steps on the wavelet tree until we arrive at the leaf node corresponding to $c$, and the current value of $i$ is the answer.

As an example, let us solve $Occ_a(S, 5)$ on the wavelet tree in Supplementary Figure 1. In $node1$, symbol 'a' belongs to the first part of alphabet, thus we compute $i \leftarrow \text{rank}_0(\mathcal{B}_{node1}, 5) = 2$ and move to the left child. In $node2$, symbol 'a' belongs to the first part of alphabet, we recompute $i \leftarrow \text{rank}_0(\mathcal{B}_{node2}, 2) = 1$ and continue on the left child. In $node4$, symbol 'a' belongs to the second part of alphabet, we recompute $i \leftarrow \text{rank}_1(\mathcal{B}_{node4}, 1) = 1$ and move to the right child. Finally, since $node4$ is a leaf node of symbol 'a', we return the current value $i = 1$.

**Algorithm 2:** Count-Pan($\mathcal{P}$)

1 $\mathcal{S} \leftarrow \{[0, n-1]\}$
2 **for** $i \leftarrow m$ **downto** 1 **do**
3     $\mathcal{S}' \leftarrow \emptyset,\ \gamma \leftarrow \mathcal{P}[i-1],\ \phi \leftarrow \Gamma_\gamma$
4     **foreach** $[l, r] \in \mathcal{S}$ **do**
5         $\mathcal{A}_l \leftarrow Occ_\phi(\mathcal{W}, l),\ \mathcal{A}_r \leftarrow Occ_\phi(\mathcal{W}, r)$
6         **foreach** $c \in \phi$ **do**
7             $l \leftarrow \mathcal{C}[c] + \mathcal{A}_l[c],\ r \leftarrow \mathcal{C}[c] + \mathcal{A}_r[c] - 1$
8             **if** $l \leq r$ **then**
9                 $\mathcal{S}' \leftarrow \mathcal{S}' \cup \{[l, r]\}$
10     $\mathcal{S} \leftarrow \mathcal{S}'$
11 **return** $\mathcal{S}$

**Supplementary Algorithm 2**

Algorithm 2 gives the pseudocode of *count* query on pangenome, given the pattern $\mathcal{P}$, it returns the corresponding SA interval set. We use the running example for $\mathcal{P} = TAA$ to show how Supplementary Algorithm 2 works for the text string $\mathcal{T}$ shown in Figure 2(c). The following process is based on Supplementary Table 2. We initialize the set $\mathcal{S}$ to be $\{[0, 40]\}$ in line 1. Next, in the outer **for** loop of lines 2–10, the base $\gamma$ taken from the pattern is $\mathcal{P}[m-1] = \mathcal{P}[2] = A$, and its corresponding IUPAC code subset $\Gamma_A = \{M, H, V, R, D, W, A\}$; as shown in lines 4 and 6, we perform interval updates for each symbol in $\Gamma_A$ based on the current SA interval set $\mathcal{S}$. The intervals for symbol $M$, $H$, $V$, $R$, and $D$ are empty (i.e., $l > r$), the interval for symbol $W$ is $[39, 39]$, and the interval for symbol $A$ is $[3, 11]$. Then we add those non-empty intervals to the temporary set $\mathcal{S}'$. In the second loop, the base $\gamma$ taken from the pattern is also $A$, and $\mathcal{S} = \{[39, 39], [3, 11]\}$ at this moment, we perform interval updates for each symbol in $\Gamma_A$ based on set $\mathcal{S}$. Among them, for [39, 39], the intervals for all symbols in $\Gamma_A$ are empty; and for [3, 11], the intervals for symbol $M$, $H$, $V$, $R$, and $D$ are empty, the interval for symbol $W$ is $[39, 39]$, and the interval for symbol $A$ is $[3, 5]$. Likewise, we add those non-empty intervals to the set $\mathcal{S}'$. In the third loop, the base $\gamma$ from the pattern is $T$, and $\mathcal{S} = \{[39, 39], [3, 5]\}$ at this moment, we repeat those steps to get $\mathcal{S} = \{[38, 38], [34, 36]\}$.

**Algorithm 3:** $Occ_\phi(\mathcal{W}, i)$

$Wt_l$ and $Wt_r$ denote the wavelet tree of $\mathcal{W}_l$ and $\mathcal{W}_r$.

$\mathcal{A} \leftarrow \emptyset$
$u \leftarrow \text{rank}_1(\mathcal{U}, i)$
**if** $\phi \cap \Sigma_{poly} \neq \emptyset$ **then**
    $\mathcal{A} \leftarrow \mathcal{A} \cup \text{accWt}(u,\ Wt_r)$
**if** $\phi \cap \Sigma_{uniq} \neq \emptyset$ **then**
    $\mathcal{A} \leftarrow \mathcal{A} \cup \text{accWt}(i - u,\ Wt_l)$
**return** $\mathcal{A}$

**procedure** accWt($i$, $Wt$)
    $\mathcal{A}' \leftarrow \emptyset$
    Enqueue($\mathcal{Q}, \langle i,\ Wt.root \rangle$)
    **while** $\mathcal{Q} \neq \emptyset$ **do**
        $\langle i, node \rangle \leftarrow$ Dequeue($\mathcal{Q}$)
        $R_1 \leftarrow \text{rank}_1(node.\mathcal{B}, i),\ R_0 \leftarrow i - R_1$
        **foreach** $\mathcal{N} \in node.Child$ **do**
            **if** $\mathcal{N}.isLeaf$ **then**
                $\mathcal{A}' \leftarrow \mathcal{A}' \cup (\mathcal{N}.isleft\ ?\ R_0 : R_1)$
            **else**
                **if** $\mathcal{N}.isleft$ **then**
                    **if** $\phi \cap node.\Sigma_l \neq \emptyset$ **then**
                        Enqueue($\mathcal{Q}, \langle R_0, \mathcal{N} \rangle$)
                **else**
                    **if** $\phi \cap node.\Sigma_r \neq \emptyset$ **then**
                        Enqueue($\mathcal{Q}, \langle R_1, \mathcal{N} \rangle$)
    **return** $\mathcal{A}'$

**Supplementary Algorithm 3**

**Algorithm 4:** Locate-Pan($\mathcal{S}$)

$\mathcal{S}$ denotes the set of SA intervals of $\mathcal{P}$.

1 $Ans \leftarrow \emptyset$<br>
2 **foreach** $[l, r] \in \mathcal{S}$ **do**<br>
3     **for** $i \leftarrow l$ **to** $r$ **do**<br>
4         $Ans \leftarrow Ans \cup \text{ChrPos}(i)$<br>
5 **return** $Ans$

**procedure** ChrPos($i$)<br>
1     $k \leftarrow 0$<br>
2     **while** $i \bmod d \neq 0$ **do**<br>
3         $k \leftarrow k + 1,\ \ c \leftarrow \mathcal{W}[i]$<br>
4         $i \leftarrow LF(i)$<br>
5     $p \leftarrow SA_l[i/d] + k$<br>
6     **foreach** $\mathcal{M} \in Ann$ **do**<br>
7         **if** $p \geq \mathcal{M}.s$ **and** $p \leq \mathcal{M}.e$ **then**<br>
8             $chr \leftarrow \mathcal{M}.chr$<br>
9             $pos \leftarrow p - \mathcal{M}.s + \mathcal{M}.o$<br>
10    **return** $chr$, $pos$

**Supplementary Algorithm 4**

Algorithm 4 gives the pseudocode of *locate* query on the compressed index, which is a variant of the locate implementation [6]. It returns the chromosome ID and position where pattern $\mathcal{P}$ is located. Here $SA_l$ is the sampled suffix array, $d$ is the sampling step, and $Ann$ is the annotation information. As an auxiliary structure, each record of $Ann$ contains four elements: $chr$, $s$, $e$, and $o$, where $chr$ is the chromosome ID it is on, $s$ and $e$ are the start and end position of the fragment on the pangenome representation sequence $\mathcal{T}$, and $o$ is the start position of the fragment on the original chromosome sequence. It should be noted that the $\mathcal{W}[i]$ in line 3 of procedure ChrPos is obtained simultaneously with $Occ_c(\mathcal{W}, i)$.

We sample the suffix array as done in [6, 11, 12, 13]. For unsampled $i$, we walk on $LF$ (see the preliminaries in the manuscript) to find the nearest suffix array sampling using $SA_l$ and return $SA_l[i/d] + k$ as desired, where $k$ is the number of walking steps on $LF$.

Continuing the example $\mathcal{P} = TAA$, we do the `locate` query for the text $\mathcal{T}$ in Figure 2(c) as follows. Given the SA interval set $\mathcal{S} = \{[38, 38], [34, 36]\}$ for $\mathcal{P}$ returned by the `count` query, the `locate` query calls ChrPos to find the values of SA[38], SA[34], SA[35], and SA[36]. We use $SA[35]$ for $i = 35$ as an example to illustrate the running of ChrPos. The suffix array sampling step is $d = 4$ and $SA_l[0, 10] = [40, 34, 35, 19, 36, 12, 23, 32, 14, 9, 15]$. In the first iteration of the **while** loop, $i = 35$ and $k = 0$. Since $(35 \mod 4) \neq 0$ (that is, $i = 35$ is not a suffix array sampling), we set $k = 1$ and update $i$ to 28 by using the $LF$ mapping function (that is, $LF(35) = 28$, see Supplementary Table 2 for details). In the second iteration of the **while** loop, since $(28 \mod 4) = 0$ (find the sampling point), the **while** loop ends. According to line 5, $p = SA_l[28/4] + 1 = SA_l[7] + 1 = 33$. Next, in lines 6–10, we check the auxiliary structure of $Ann$ and find that $p$ is located in the second appended sequence, INDEL, in Figure 2(c); the chromosome ID and position of $p$ on the reference genome can then be easily retrieved. In this example, not only do we find two exact $TAA$ strings (that is, $SA[36] = 9$ and $SA[34] = 16$, see Figure 2(c) in manuscript), but we also find a $TWA$ string containing a known SNP variant (that is, $SA[38] = 3$) and a string containing a known deletion variant (that is, $SA[35] = 33$). This reflects the accuracy advantage of using pangenome index for genomic sequence analysis.

**Algorithm 5:** Extract-Pan($pos, \ell$)

$Seq$ denotes the substring extracted from pangenome index.

$Seq[0, \ell] \leftarrow [0 \cdots 0]$
$end \leftarrow pos + \ell$
$i \leftarrow SA_l^{-1}[\lceil end/d \rceil]$
$step \leftarrow d - (end \bmod d)$
**for** $j \leftarrow 1$ **to** $step$ **do**
    $c \leftarrow \mathcal{W}[i]$
    $i \leftarrow \mathcal{C}[c] + Occ_c(\mathcal{W}, i)$
**for** $j \leftarrow 1$ **to** $\ell$ **do**
    $c \leftarrow \mathcal{W}[i]$
    $Seq[\ell - j] \leftarrow c$
    $i \leftarrow \mathcal{C}[c] + Occ_c(\mathcal{W}, i)$
**return** $Seq$

**Supplementary Algorithm 5**

Algorithm 5 gives the pseudocode of *extract* query on pangenome, which is based upon the extract algorithm [6, 8, 9]. It returns the substring with the starting position $pos$ and length $\ell$. Here $SA_l^{-1}$ is the sampled inverse suffix array [8, 9]. $d$ is the inverse suffix array sampling step.

**Algorithm 6:** findLEOF($\mathcal{R}$)

1 $maxLen \leftarrow 0$
2 $\mathcal{D}_1 \leftarrow \text{getD}(\mathcal{R}),\ \mathcal{D}_2 \leftarrow \text{getD}(\overline{\mathcal{R}'})$
3 $prev \leftarrow 0,\ d_1 \leftarrow \mathcal{D}_1[0],\ d_2 \leftarrow \mathcal{D}_2[m-1]$
4 **for** $i \leftarrow 1$ **to** $m-1$ **do**
5     **if** $d_1 \neq \mathcal{D}_1[i]$ **then**
6         $K_1 \leftarrow K_1 \cup \{i\},\ d_1 \leftarrow \mathcal{D}_1[i]$
7     **if** $d_2 \neq \mathcal{D}_2[m-i-1]$ **then**
8         $K_2 \leftarrow K_2 \cup \{i\},\ d_2 \leftarrow \mathcal{D}_2[m-i-1]$
9 $K \leftarrow \text{merge}(K_1, K_2)$
10 **foreach** $k \in K$ **do**
11     **if** $maxLen < k - prev$ **then**
12         $maxLen \leftarrow k - prev,\ s \leftarrow prev,\ e \leftarrow k$
13     $prev \leftarrow k$
14 **return** $s,\ e$

**procedure** getD($\mathcal{P}$)

1     $z \leftarrow 0,\ \mathcal{S} \leftarrow \{[0,\ n-1]\}$
2     **for** $i \leftarrow m$ **to** $1$ **do**
3         $\mathcal{S}' \leftarrow \emptyset,\ \gamma \leftarrow \mathcal{P}[i-1],\ \phi \leftarrow \Gamma_\gamma$
4         **foreach** $[l,\ r] \in \mathcal{S}$ **do**
5             $\mathcal{A}_l \leftarrow Occ_\phi(\mathcal{W}, l),\ \mathcal{A}_r \leftarrow Occ_\phi(\mathcal{W}, r)$
6             **foreach** $c \in \phi$ **do**
7                 $l \leftarrow \mathcal{C}[c] + \mathcal{A}_l[c],\ r \leftarrow \mathcal{C}[c] + \mathcal{A}_r[c] - 1$
8                 **if** $l \leq r$ **then**
9                     $\mathcal{S}' \leftarrow \mathcal{S}' \cup \{[l,\ r]\}$
10         **if** $\mathcal{S}' = \emptyset$ **then**
11             $z \leftarrow z + 1,\ \mathcal{S}' \leftarrow \{[0,\ n-1]\}$
12         $\mathcal{S} \leftarrow \mathcal{S}',\ \mathcal{D}[i-1] \leftarrow z$
13     **return** $\mathcal{D}$

**Supplementary Algorithm 6**

Algorithm 6 describes the pseudocode of finding the LEOF, where $m$ is the length of the read sequence, $n$ is the length of pangenome representation sequence. In line 2, we input the forward read sequence $\mathcal{R}$ and its reverse complement $\overline{\mathcal{R}'}$, and use *getD* function to calculate the $\mathcal{D}_1$ and $\mathcal{D}_2$ arrays. In lines 4–8, we find the locations of discontinuity points in the two $\mathcal{D}$ arrays and save them in set $K_1$ and $K_1$ respectively. Then, in lines 9–14, the locations in two sets are merged and sorted, and the starting position $s$ and ending position $e$ of the maximum interval on read are returned. For the *getD* function, it is a variation of the `count` query on the pangenome. As shown in `procedure` line 2, we perform interval updates along read from back to front until the interval becomes empty. At this point, we increment the variable $z$ by one and reset the interval to its initial state (see `procedure` lines 10-11). For each base in read, we save the value of variable $z$ in $\mathcal{D}$ array in `procedure` line 12. Taking $\mathcal{D}_1$ in Figure 4 as an example, we perform interval updates along read and increment the variable $z$ by one when the interval becomes empty at locus 19. After resetting the interval, we continue to update along read and increment $z$ when the interval becomes empty again at locus 38. Similarly, the next time interval becomes empty occurs at locus 93.

Table 1: Compact structure for bitvector $\mathcal{U}$.

| $\mathcal{W}$ | T | C | C | T | T | T | A | T | A | W | # | A | A | C | C | A | A | A | A | $ | A | C | # | C | C | C | C | C | C | C | C | C | C | C | Y | C | C | C | C | T | C |
|---|---|---|---|---|---|---|---|---|---|---|---|---|---|---|---|---|---|---|---|---|---|---|---|---|---|---|---|---|---|---|---|---|---|---|---|---|---|---|---|---|---|
| $\mathcal{U}$ | 0 | 0 | 0 | 0 | 0 | 0 | 0 | 0 | 0 | 1 | 1 | 0 | 0 | 0 | 0 | 0 | 0 | 0 | 0 | 1 | 0 | 0 | 1 | 0 | 0 | 0 | 0 | 0 | 0 | 0 | 0 | 0 | 0 | 0 | 1 | 0 | 0 | 0 | 0 | 0 | 0 |
| *Brank* | 0 | | | | | 0 | | | | | 1 | | | | | 0 | | | | | 1 | | | | | 2 | | | | | 0 | | | | | 1 | | | | | |
| *SBrank* | 0 | | | | | | | | | | | | | | | 2 | | | | | | | | | | | | | | | 4 | | | | | | | | | | |
| *Bpos* | | | | | | 4 | | | | | 0 | | | | | 4 | | | | | 2 | | | | | | | | | | 4 | | | | | | | | | | |
| *Bmark* | 0 | | | | | 1 | | | | | 1 | | | | | 1 | | | | | 1 | | | | | 0 | | | | | 1 | | | | | 0 | | | | | |

**Supplementary Table 1**

Table 1 shows the structures for the representation of bitvector $\mathcal{U}$. This representation for the bit vector borrows the compression structures for the bit strings of the wavelet tree nodes [6, 7, 9]. We partition $\mathcal{U}$ into blocks of size $b$ and combine contiguous blocks to form superblocks of size $sb$, where $b = 5$ and $sb = 15$ in this example. In practice, the block size $b = 1024$ and the superblock size $sb = 16 \times b$. Next, we construct four fixed-length arrays named $Brank$, $SBrank$, $Bpos$, and $Bmark$, where $Brank$ stores the number of 1s in $\mathcal{U}$ preceding the current block relative to the beginning of its enclosing superblock; $SBrank$ stores the number of 1s in $\mathcal{U}$ preceding the current superblock; $Bpos$ stores the relative position of 1s within the block enclosing it; $Bmark$ stores the bit mark of whether the current block contains 1s. To answer the $\mathrm{rank}_1(\mathcal{U}, i)$ queries, we compute it as $\mathrm{rank}_1(\mathcal{U}, i) = SBrank[i/sb] + Brank[i/b] + \mathrm{rankInB}(\mathit{offset}, Bmark, Bpos)$ where $\mathit{offset} = i \bmod b$. Here the rankInB function returns the number of 1s up to position $\mathit{offset}$ within the last block, there are four cases: (1) when $Bmark[i/b] = 0$, i.e., the last block does not contain 1s, it returns the $value$ of 0 directly; (2) when $Bmark[i/b] = 1$ and $Bpos[\cdot] < \mathit{offset}$, i.e., the current 1s in the block has not yet reached $i$, let $value \leftarrow value + 1$ and check the next 1s in the block; (3) when $Bmark[i/b] = 1$ and $Bpos[\cdot] = \mathit{offset}$, i.e., the current 1s in the block is exactly at $i$, let $value \leftarrow value + 1$ and return it; (4) when $Bmark[i/b] = 1$ and $Bpos[\cdot] > \mathit{offset}$, that is, the current 1s in the block has jumped over $i$, hence it should return the current $value$.

Table 2: The Burrows-Wheeler transform for text $\mathcal{T}$ shown in Figure 2(c).

| $i$ | $\mathcal{T}[i]$ | $SA(i)$ | $SA^{-1}(i)$ | $LF(i)$ | $F$ $M$ $L$ |
|---|---|---|---|---|---|
| 0 | C | 40 | 19 | 33 | $CCCTWACCCTAACCCYTAAC#ACCCTACCC#CCTAACCCT |
| 1 | C | 20 | 25 | 12 | #ACCCTACCC#CCTAACCCT$CCCTWACCCTAACCCYTAAC |
| 2 | C | 30 | 31 | 13 | #CCTAACCCT$CCCTWACCCTAACCCYTAAC#ACCCTACCC |
| 3 | T | 17 | 38 | 34 | AAC#ACCCTACCC#CCTAACCCT$CCCTWACCCTAACCCYT |
| 4 | W | 34 | 39 | 35 | AACCCT$CCCTWACCCTAACCCYTAAC#ACCCTACCC#CCT |
| 5 | A | 10 | 9 | 36 | AACCCYTAAC#ACCCTACCC#CCTAACCCT$CCCTWACCCT |
| 6 | C | 18 | 17 | 3 | AC#ACCCTACCC#CCTAACCCT$CCCTWACCCTAACCCYTA |
| 7 | C | 26 | 23 | 37 | ACCC#CCTAACCCT$CCCTWACCCTAACCCYTAAC#ACCCT |
| 8 | C | 35 | 29 | 4 | ACCCT$CCCTWACCCTAACCCYTAAC#ACCCTACCC#CCTA |
| 9 | T | 5 | 36 | 39 | ACCCTAACCCYTAAC#ACCCTACCC#CCTAACCCT$CCCTW |
| 10 | A | 21 | 5 | 1 | ACCCTACCC#CCTAACCCT$CCCTWACCCTAACCCYTAAC# |
| 11 | A | 11 | 11 | 5 | ACCCYTAAC#ACCCTACCC#CCTAACCCT$CCCTWACCCTA |
| 12 | C | 19 | 20 | 6 | C#ACCCTACCC#CCTAACCCT$CCCTWACCCTAACCCYTAA |
| 13 | C | 29 | 26 | 14 | C#CCTAACCCT$CCCTWACCCTAACCCYTAAC#ACCCTACC |
| 14 | C | 28 | 32 | 15 | CC#CCTAACCCT$CCCTWACCCTAACCCYTAAC#ACCCTAC |
| 15 | Y | 27 | 40 | 7 | CCC#CCTAACCCT$CCCTWACCCTAACCCYTAAC#ACCCTA |
| 16 | T | 36 | 34 | 8 | CCCT$CCCTWACCCTAACCCYTAAC#ACCCTACCC#CCTAA |
| 17 | A | 6 | 3 | 9 | CCCTAACCCYTAAC#ACCCTACCC#CCTAACCCT$CCCTWA |
| 18 | A | 22 | 6 | 10 | CCCTACCC#CCTAACCCT$CCCTWACCCTAACCCYTAAC#A |
| 19 | C | 0 | 12 | 0 | CCCTWACCCTAACCCYTAAC#ACCCTACCC#CCTAACCCT$ |
| 20 | # | 12 | 1 | 11 | CCCYTAAC#ACCCTACCC#CCTAACCCT$CCCTWACCCTAA |
| 21 | A | 37 | 10 | 16 | CCT$CCCTWACCCTAACCCYTAAC#ACCCTACCC#CCTAAC |
| 22 | C | 31 | 18 | 2 | CCTAACCCT$CCCTWACCCTAACCCYTAAC#ACCCTACCC# |
| 23 | C | 7 | 24 | 17 | CCTAACCCYTAAC#ACCCTACCC#CCTAACCCT$CCCTWAC |
| 24 | C | 23 | 30 | 18 | CCTACCC#CCTAACCCT$CCCTWACCCTAACCCYTAAC#AC |
| 25 | T | 1 | 37 | 19 | CCTWACCCTAACCCYTAAC#ACCCTACCC#CCTAACCCT$C |
| 26 | A | 13 | 7 | 20 | CCYTAAC#ACCCTACCC#CCTAACCCT$CCCTWACCCTAAC |
| 27 | C | 38 | 15 | 21 | CT$CCCTWACCCTAACCCYTAAC#ACCCTACCC#CCTAACC |
| 28 | C | 32 | 14 | 22 | CTAACCCT$CCCTWACCCTAACCCYTAAC#ACCCTACCC#C |
| 29 | C | 8 | 13 | 23 | CTAACCCYTAAC#ACCCTACCC#CCTAACCCT$CCCTWACC |
| 30 | # | 24 | 2 | 24 | CTACCC#CCTAACCCT$CCCTWACCCTAACCCYTAAC#ACC |
| 31 | C | 2 | 22 | 25 | CTWACCCTAACCCYTAAC#ACCCTACCC#CCTAACCCT$CC |
| 32 | C | 14 | 28 | 26 | CYTAAC#ACCCTACCC#CCTAACCCT$CCCTWACCCTAACC |
| 33 | T | 39 | 35 | 27 | T$CCCTWACCCTAACCCYTAAC#ACCCTACCC#CCTAACCC |
| 34 | A | 16 | 4 | 40 | TAAC#ACCCTACCC#CCTAACCCT$CCCTWACCCTAACCCY |
| 35 | A | 33 | 8 | 28 | TAACCCT$CCCTWACCCTAACCCYTAAC#ACCCTACCC#CC |
| 36 | C | 9 | 16 | 29 | TAACCCYTAAC#ACCCTACCC#CCTAACCCT$CCCTWACCC |
| 37 | C | 25 | 21 | 30 | TACCC#CCTAACCCT$CCCTWACCCTAACCCYTAAC#ACCC |
| 38 | C | 3 | 27 | 31 | TWACCCTAACCCYTAAC#ACCCTACCC#CCTAACCCT$CCC |
| 39 | T | 4 | 33 | 38 | WACCCTAACCCYTAAC#ACCCTACCC#CCTAACCCT$CCCT |
| 40 | $ | 15 | 0 | 32 | YTAAC#ACCCTACCC#CCTAACCCT$CCCTWACCCTAACCC |

**Supplementary Table 2**

Table 2 gives the Burrows-Wheeler transform of the pangenome representation sequence $\mathcal{T}$ = CCCTWACCCTAAC CCYTAAC#ACCCTACCC#CCTAACCCT in Figure 2(c), where the first column is the ranking, the second column is $\mathcal{T}$, the third column is the unsampled suffix array, the fourth column is the unsampled inverse suffix array, the fifth column is the result of the current ranking after once $LF$ mapping, and the last column is the Burrows-Wheeler matrix, where the underlined part is the sorted suffix $\mathcal{T}[SA(i), n]$, and $F$ and $L$ indicate the first and last characters respectively. Additionally, the beginning of suffixes matching the pattern $\mathcal{P} = TAA$ are marked with a gray background.

Table 3: Commands of comparison methods used in the experiment

| Method | Command |
|---|---|
| BWBBLE | ./bwbble align -n {$k$-difference} {*index*} {*reads*} {*aln*}<br>./bwbble aln2sam {*index*} {*reads*} {*aln*} {*sam*} |
| HISAT2 | ./hisat2 --no-spliced-alignment -x {*index*} -U {*reads*} -S {*sam*} |
| Bowtie2 | ./bowtie2 -x {*index*} -U {*reads*} -S {*sam*} |
| BWA-MEM | ./bwa mem {*index*} {*reads*} > {*sam*} |
| BWA-MEM2 | ./bwa-mem2 mem {*index*} {*reads*} > {*sam*} |
| VG | ./vg map -f {*reads*} -x {*index_xg*} -g {*index_gcsa*} --surject-to bam > {*bam*} |
| Giraffe | ./vg giraffe -Z {*index*} -f {*reads*} -o BAM > {*bam*} |
| GraphAligner | ./GraphAligner -g {*graph_vg*} -f {*reads*} -a {*gam*} -x vg |
| Minichain | ./minichain -cx lr {*graph_gfa*} {*reads*} > {*gaf*} |

Note: To be comparable with other methods, the default $k$-difference is set to 3.

**Supplementary Table 3**

Table 4: Space usage of each index structure in Panaln

| Structure | Space usage | Description | Parameters |
|---|---|---|---|
| $\mathcal{U}$ | 0.058 GB | Compressed bitvector at the root | Block size $b$ is 1024, superblock size $sb$ is $b \times 16$ |
| $Wt_l$ | 1.829 GB | Balanced wavelet tree of the left subtree | Block size of the bitvector on nodes is 256 |
| $Wt_r$ | 0.012 GB | Huffman-shaped wavelet tree of the right subtree | Block size of the bitvector on nodes is 256 |
| $\mathcal{Z}$ | 0.285 GB | Sampling mark array | Block size $b$ is 256, superblock size $sb$ is $b \times 16$ |
| $SA_l$ | 0.754 GB | Sampled suffix array | Sampling rate is 32 |
| $SA_l^{-1}$ | 0.754 GB | Sampled inverse suffix array | Sampling rate is 32 |

Note: Here pangenome is constructed from the whole genome (GRCh38) and variants in dbSNP (v144).

**Supplementary Table 4**

Table 5: SNV and INDEL calling with bcftools on GiaB dataset

| **SNV** | $F$-score | Precision | Recall | TP | P |
|---|---|---|---|---|---|
| Panaln | 0.087342 | 0.756815 | 0.046346 | 160,062 | 211,494 |
| BWBBLE | 0.084447 | 0.758849 | 0.044712 | 154,419 | 203,491 |
| HISAT2 | 0.087312 | 0.717187 | 0.046486 | 160,546 | 223,855 |
| Bowtie2 | 0.080335 | 0.796764 | 0.042301 | 146,090 | 183,354 |
| BWA-MEM | 0.086431 | 0.755239 | 0.045839 | 158,311 | 209,617 |
| BWA-MEM2 | 0.086431 | 0.755239 | 0.045839 | 158,311 | 209,617 |
| **INDEL** | $F$-score | Precision | Recall | TP | P |
| Panaln | 0.000249 | 0.120000 | 0.000130 | 75 | 625 |
| BWBBLE | 0.000178 | 0.246511 | 0.000091 | 53 | 215 |
| HISAT2 | 0.000227 | 0.100890 | 0.000117 | 68 | 674 |
| Bowtie2 | 0.000223 | 0.102603 | 0.000115 | 67 | 653 |
| BWA-MEM | 0.000246 | 0.097428 | 0.000124 | 72 | 739 |
| BWA-MEM2 | 0.000246 | 0.097428 | 0.000124 | 72 | 739 |

**Supplementary Table 5**

In the experiments, we follow the pipeline described in previous work [17] and use bcftools to benchmark the $F$-score, precision, and recall of SNV and INDEL calling over each aligner's output on real Illumina reads provided by the GiaB consortium. The poor results of all tools are due to the low coverage of the reads dataset. Considering the long execution time of bcftool, we use the first one million subset of the full dataset with $17\times$ coverage, which produces lower recall and thus lower $F$-score. As mentioned in the literature [19], higher coverage can produce better variant calls, however the reads coverage in this experiment is fair and unbiased for all compared methods.

## Supplementary Figure 1

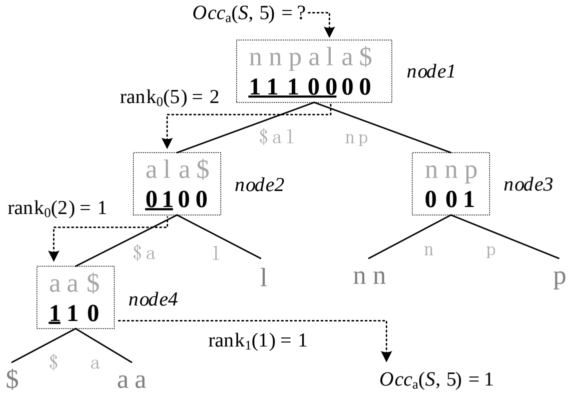


Figure 1: The wavelet tree for the BWT string of $\mathcal{T} = panaln$.

An example of the balanced wavelet tree is shown in Figure 1. Here alphabet $\Sigma = \{\$, \text{a}, \text{l}, \text{n}, \text{p}\}$, it is divided into two parts $\{\$, \text{a}, \text{l}\}$ and $\{\text{n}, \text{p}\}$ according to the lexicographical order. Thus, the symbols belonging to the former part of the root node *node1* are assigned to the left child *node2*, and the symbols belonging to the other part are assigned to the right child of *node3*. Similarly, the node of *node2* produces its left and right children nodes, where the right child is a leaf node because it contains a single symbol 'l'. Note that we store only the bold elements of the bit vector within each node; and the light gray character string is provided solely for conceptual illustration.

## Supplementary Figure 2

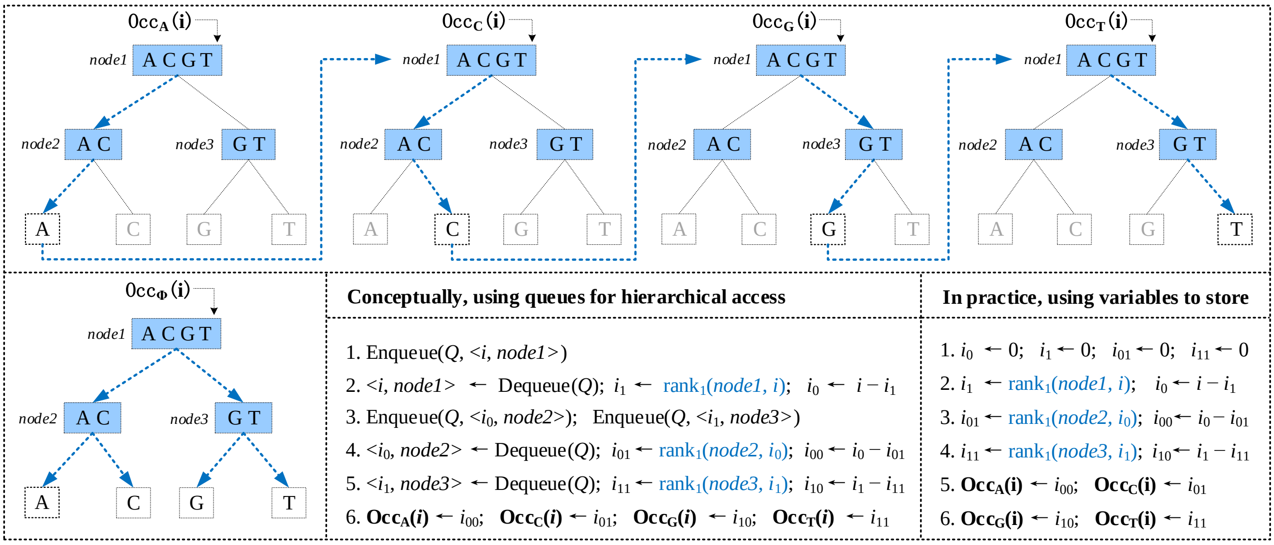


Figure 2: Illustration of batching query method $Occ_\phi(\mathcal{W}, i)$.

Conceptually, we introduce a queue to hierarchically access the nodes of the wavelet tree. However, as shown in Supplementary Figure 2, the fixed structure of the wavelet tree allows us to customize the access path based on the assigned symbol set for efficiency, thereby avoiding the overhead of queue operations in practice. For example, when given $\phi = \{\text{A}, \text{C}, \text{G}, \text{T}\}$, the number of access and $\text{rank}_b(\mathcal{B}, i)$ operations of *node1* in Supplementary Figure 2 would be reduced from 4 with $Occ_c(\mathcal{W}, i)$ where $c \in \phi$ to 1 with our $Occ_\phi(\mathcal{W}, i)$. Similarly, the number of access and $\text{rank}_b(\mathcal{B}, i)$ operations for *node2* and *node3* would be reduced from 2 to 1. Furthermore, the higher the common ancestor node, the more times it is reused. Therefore, $Occ_\phi(\mathcal{W}, i)$ performs better on Huffman-shaped wavelet tree.

**Supplementary Figure 3**

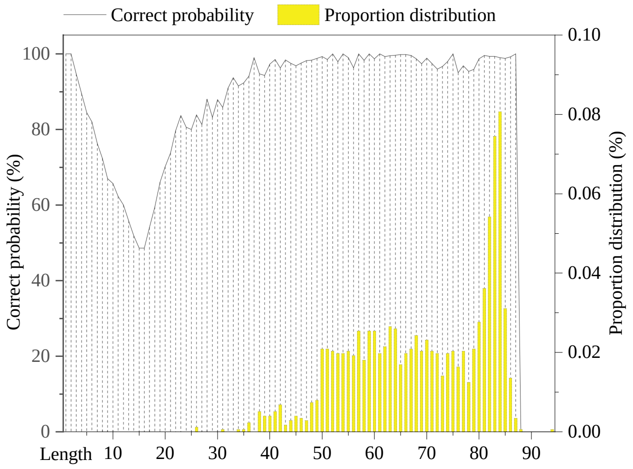


Figure 3: Correct probability of EOFs and proportion distribution of reads with specific LEOF length on 10k simulated reads with the known source locations and true alignments.

To evaluate the effectiveness of LEOF, we tested on ten thousand Illumina-like 101-bp simulated reads whose source positions and alignments are known. Supplementary Figure 3 provides the correct probability of EOFs and the proportion distribution of reads with a specific LEOF length, where the x-axis is fragment length, the left y-axis is the correct probability of EOFs checked to contain no sequencing errors or unknown variants, and the right y-axis is the proportion distribution of reads with a specific LEOF length. We can see that the correct probability shows a $V$-shaped curve as the length of EOFs increases, and the inflection point occurs at length 15. The higher correct probability at the beginning of the curve is because EOFs length is short, thus the occurrence probability of variants and sequencing errors is relatively low. The increase in the subsequent curve is due to the longer EOFs sequences making it more sensitive to variants and sequencing errors. In particular, we observe that almost all LEOFs are distributed in the stable region with high correct probability. Therefore, the experiment verified its effectiveness.

## Data Availability

- Illumina reads dataset: https://ftp-trace.ncbi.nlm.nih.gov/ReferenceSamples/giab/data_indexes/AshkenazimTrio/sequence.index.AJtrio_Illumina_2x250bps_06012016_updated.HG004
- PacBio-CCS reads dataset: https://ftp-trace.ncbi.nlm.nih.gov/ReferenceSamples/giab/data/AshkenazimTrio/HG002_NA24385_son/PacBio_CCS_10kb/m54238_180628_014238.Q20.fastq
- Common small variants: http://hgdownload.soe.ucsc.edu/goldenPath/hg38/database/snp144Common.txt.gz
- Synthetic reads simulator: https://github.com/DaehwanKimLab/hisat2/blob/master/hisat2_simulate_reads.py
- Pipeline of variant calling: https://github.com/ksahlin/strobealign/blob/main/evaluation.md
- Source code: https://github.com/Lilu-guo/PanaIn
- Software package: https://anaconda.org/bioconda/panaln
- Small example data for testing: https://github.com/Lilu-guo/PanaIn/tree/master/Test

## References


[1] Stefan Canzar and Steven L Salzberg. Short read mapping: an algorithmic tour. *Proceedings of the IEEE*, 105(3):436–458, 2017.

[2] Erik Garrison, Jouni Sirén, Adam M Novak, Glenn Hickey, Jordan M Eizenga, Eric T Dawson, William Jones, Shilpa Garg, Charles Markello, Michael F Lin, et al. Variation graph toolkit improves read mapping by representing genetic variation in the reference. *Nature biotechnology*, 36(9):875–879, 2018.

[3] Roberto Grossi, Ankur Gupta, and Jeffrey Scott Vitter. High-order entropy-compressed text indexes. In *Proc. of 14th ACM-SIAM Symposium on Discrete Algorithms*, pages 841–850, 2003.

[4] Roberto Grossi, Jeffrey Scott Vitter, and Bojian Xu. Wavelet trees: From theory to practice. In *2011 First International Conference on Data Compression, Communications and Processing*, pages 210–221. IEEE, 2011.

[5] Lin Huang, Victoria Popic, and Serafim Batzoglou. Short read alignment with populations of genomes. *Bioinformatics*, 29(13): i361–i370, 2013.

[6] Hongwei Huo, Longgang Chen, Heng Zhao, Jeffrey Scott Vitter, Yakov Nekrich, and Qiang Yu. A data-aware FM-Index. In *2015 Proceedings of the Seventeenth Workshop on Algorithm Engineering and Experiments (ALENEX)*, pages 10–23. SIAM, 2015.

[7] Hongwei Huo, Pengfei Liu, Chenhui Wang, Hongbo Jiang, and Jeffrey Scott Vitter. CIndex: compressed indexes for fast retrieval of FASTQ files. *Bioinformatics*, 38(2):335–343, 2022.

[8] Hongwei Huo, Peng Long, and Jeffrey Scott Vitter. Practical high-order entropy-compressed text self-indexing. *IEEE Transactions on Knowledge and Data Engineering*, 35(3):2943–2960, 2023.

[9] Hongwei Huo, Zongtao He, Pengfei Liu, and Jeffrey Scott Vitter. FM-Adaptive: A practical data-aware FM-Index. In *The Expanding World of Compressed Data 2025*, page 5:1–5:23. OASIcs, Schloss Dagstuhl, Leibniz-Zentrum für Informatik, 2025.

[10] Z Iqbal, S Maciuca, C del Ojo Elias, and G McVean. A natural encoding of genetic variation in a burrows-wheeler transform to enable mapping and genome inference. In *Workshop on Algorithms in Bioinformatic*. Springer, 2016.

[11] Daehwan Kim, Joseph M Paggi, Chanhee Park, Christopher Bennett, and Steven L Salzberg. Graph-based genome alignment and genotyping with hisat2 and hisat-genotype. *Nature biotechnology*, 37(8):907–915, 2019.

[12] Ben Langmead, Cole Trapnell, Mihai Pop, and Steven L Salzberg. Ultrafast and memory-efficient alignment of short dna sequences to the human genome. *Genome biology*, 10(3):1–10, 2009.

[13] Heng Li and Richard Durbin. Fast and accurate short read alignment with burrows–wheeler transform. *bioinformatics*, 25(14): 1754–1760, 2009.

[14] Heng Li, Xiaowen Feng, and Chong Chu. The design and construction of reference pangenome graphs with minigraph. *Genome biology*, 21:1–19, 2020.

[15] Giovanni Manzini. An analysis of the burrows—wheeler transform. *Journal of the ACM (JACM)*, 48(3):407–430, 2001.

[16] Mikko Rautiainen and Tobias Marschall. Graphaligner: rapid and versatile sequence-to-graph alignment. *Genome biology*, 21(1): 253, 2020.

[17] Kristoffer Sahlin. Strobealign: flexible seed size enables ultra-fast and accurate read alignment. *Genome Biology*, 23(1):260, 2022.

[18] Kristoffer Sahlin, Thomas Baudeau, Bastien Cazaux, and Camille Marchet. A survey of mapping algorithms in the long-reads era. *Genome Biology*, 24(1):133, 2023.

[19] Jouni Sirén, Jean Monlong, Xian Chang, Adam M Novak, Jordan M Eizenga, Charles Markello, Jonas A Sibbesen, Glenn Hickey, Pi-Chuan Chang, Andrew Carroll, et al. Pangenomics enables genotyping of known structural variants in 5202 diverse genomes. *Science*, 374(6574):abg8871, 2021.
